# Serum Immunoglobulins, Pneumonia Risk, and Lung Function in Middle-Aged and Older Individuals: A Population-Based Cohort Study

**DOI:** 10.3389/fimmu.2022.868973

**Published:** 2022-06-02

**Authors:** Samer R. Khan, Anna Vanoverschelde, Lies Lahousse, Robin P. Peeters, P. Martin van Hagen, Guy Brusselle, Layal Chaker, Virgil A. S. H. Dalm

**Affiliations:** ^1^ Department of Internal Medicine, Division of Allergy and Clinical Immunology, Erasmus University Medical Center, Rotterdam, Netherlands; ^2^ Department of Epidemiology, Erasmus University Medical Center, Rotterdam, Netherlands; ^3^ Department of Bioanalysis, Pharmaceutical Care Unit, Ghent University, Ghent, Belgium; ^4^ Department of Internal Medicine, Division of Endocrinology, Erasmus University Medical Center, Rotterdam, Netherlands; ^5^ Department of Immunology, Erasmus University Medical Center, Rotterdam, Netherlands; ^6^ Department of Respiratory Medicine, Ghent University Hospital, Ghent, Belgium; ^7^ Department of Respiratory Medicine, Erasmus University Medical Center, Rotterdam, Netherlands

**Keywords:** pneumonia, immunoglobulin A, immunoglobulin G, immunoglobulin M, pulmonary ventilation

## Abstract

**Introduction:**

Immunoglobulins (Igs) play a pivotal role in host defense and prevention of pneumonia. Aging influences serum Ig levels, but the association between Igs and pneumonia in community-dwelling older individuals remains unknown. We evaluated the association of serum IgA, IgG, and IgM with pneumonia and lung function in middle-aged and older individuals.

**Methods:**

We performed Cox and negative binomial regression analyses for the association of Igs with incident pneumonia and pneumonia-related mortality, and recurrent pneumonia respectively. We performed logistic regression analyses for the association between Igs and lung function values. Associations were adjusted for age, sex, smoking, comorbidities, and serum C-reactive protein.

**Results:**

We included 8,766 participants (median age 62.2 years, 57% women, median follow-up 9.8 years). Higher IgA (hazard ratio [HR]: 1.15; 95% confidence interval [95% CI]: 1.00-1.32) and IgG (HR: 1.13; 95% CI: 1.06-1.19) were associated with an increased pneumonia risk. Higher IgG was associated with an increased risk of pneumonia-related mortality (HR: 1.08; 95% CI: 1.01-1.16) and recurrent pneumonia (incidence rate ratio: 1.04; 95% CI: 1.00-1.09). Higher IgA and IgG were also associated with lower forced expiratory volume in one second (FEV_1_), lower forced vital capacity (FVC), and an increased odds of preserved ratio impaired spirometry (PRISm, i.e. FEV_1_ <80% and FEV_1_/FVC ratio ≥70%). No association was seen with an obstructive spirometry pattern.

**Discussion:**

Higher serum IgA and IgG levels were associated with pneumonia, pneumonia-related mortality, and PRISm in middle-aged and older individuals from the general population. Future studies should validate our findings and elucidate underlying pathophysiology.

## Introduction

An adequate immune system with equilibrium between pro- and anti-inflammatory responses is pivotal for prevention of pneumonia (1). Primary antibody deficiencies are characterized by low levels of serum immunoglobulins (Igs) and increased susceptibility to upper and lower respiratory tract infections, including pneumonia (2). Immunoglobulin replacement therapy (IgRT) decreases the incidence and severity of pneumonia in these patients and is associated with a better forced expiratory volume in one second (FEV_1_) (3–5). A higher trough IgG level after IgRT is associated with a decrease in risk of developing pneumonia and a decrease in lung function decline (6, 7).

However, in individuals without primary immunodeficiencies, higher levels of Igs may also be associated with the risk of developing pneumonia. Chronic inflammation or chronic inflammatory conditions that are associated with increased serum Ig levels, have been associated with pneumonia susceptibility as well (8). The direction of the association between Ig levels and risk of infections could therefore depend on underlying disease or cause.

Aging is an important risk factor for developing pneumonia due to impaired immune responses amongst others. Age-related chronic inflammation has been associated with increased pathogen adhesion and immune dysregulation (9). In a previous study of 39 healthy volunteers, older compared to younger individuals had higher Ig levels in the bronchoalveolar fluid and lower predicted lung function values, although the latter did not reach statistical significance (10). Recently, we have shown age-related increases of serum IgA and IgG in our general population cohort of middle-aged and older individuals (11). It is unclear whether an association exists of serum Igs with pneumonia or impaired lung function in this older population. Uncovering novel risk factors of pneumonia at older age is of timely importance given the highest prevalence (number of cases at a specific time point) and incidence (number of new cases developing over a specific period of time) of pneumonia and obstructive or restrictive spirometry patterns in this age group.

Previous studies on the association of serum Ig levels with pneumonia prevalence and severity in immunocompetent individuals reported inconclusive results and were performed in adults with a heterogeneous age range (12–16). These studies furthermore had several limitations that potentially restrict extrapolation of results to older individuals from a general population. Previous studies were either cross-sectional, had small sample sizes, were patient-based, or did not account for potential confounders.

It is therefore still unknown whether and how serum Igs are associated with the risk of developing pneumonia and pneumonia-related complications in community-dwelling middle-aged and older individuals. We performed this study to assess the association of serum Ig levels with risk of pneumonia and related mortality as well as lung function in a large prospective population-based cohort.

## Methods

### Study Population

This study was embedded in the Rotterdam Study (RS), an ongoing prospective population-based cohort study of middle-aged and older participants from Ommoord, a suburb of Rotterdam, the Netherlands. The RS focuses on the epidemiology of conditions from various medical fields, including respiratory medicine and internal medicine. The RS started in 1990 and was initially aimed at inhabitants of the study area aged 55 or over. The RS was extended with additional cohorts in 2000, 2006, and 2016. For these cohorts, inhabitants of the study area that had not been previously invited and were aged 55 (RS II), 45 (RS III), or 40 years or over (RS IV), were invited to participate. The overall response rate of the RS for the first three cohorts was 72% (14,926 out of 20,744). After enrollment, participants are re-examined every 3-6 years at the research center in Ommoord. In addition, continuous linkage takes places of the research database with medical records of general practitioners (GPs) and hospitals. The RS has been approved by the medical ethics committee of Erasmus University Medical Center (registration number MEC 02.1015) and the Dutch Ministry of Health, Welfare, and Sport (Population Screening Act WBO, license number 1071272-159521-PG). Detailed information has been published elsewhere (17). For this study, we included participants from three independent RS cohorts (I-3, II-1, III-1) with available baseline measurements of serum IgA, IgG, and/or IgM, written informed consent for follow-up, and information on pneumonia.

### Assessment of Serum Igs

Venous blood was drawn at the research center in Ommoord in 1997 through 2008, and the moment of blood drawing was considered the study baseline. Serum samples were subsequently stored at -80°C and Ig levels were analyzed between 2016 and 2018 with an immunoturbidimetric assay (Tina-quant^®^ IgA/IgG/IgM Gen. 2, Roche Diagnostics GmbH, Mannheim, Germany). According to the assay manufacturer’s protocol, recommended reference ranges for adults were 0.7-4.0 g/L for IgA, 7.0-16.0 g/L for IgG, and 0.4-2.3 g/L for IgM. However, based on 2.5th and 97.5th percentiles for the entire study population with Ig measurements, we previously retrieved 0.86-4.76 g/L for IgA, 6.20-15.10 g/L for IgG, and 0.28-2.64 g/L for IgM (11).

### Assessment of Pneumonia and Lung Function

The diagnosis of prevalent (events occurring before blood drawing) and incident (events occurring after blood drawing) pneumonia was based on medical records (including hospital discharge letters) of GPs. All pneumonia events (prevalent or incident) included in this study were cases of community-acquired pneumonia (CAP). Until 2012, the national database on hospitalizations (Landelijke Medische Registratie) was used to select participants with pneumonia (ICD-9 codes 480-488) as main discharge diagnosis. Pneumonia hospitalizations that could not be verified in case of limited access to medical records were included to avoid underestimation of older pneumonias. After 2012, trained follow-up assistants collected all information mentioning ‘pneumonia’ by scrutinizing medical records at GP offices. This copied information was coded by a researcher based on medical records and chest imaging. Pneumonia cases were furthermore distinguished into definite and probable. Definite cases were defined as a clinical diagnosis of pneumonia made by a pulmonary physician or GP, suggestive clinical symptoms, and an infiltrate on chest imaging. In the absence of chest imaging, cases were regarded probable if the first two criteria were met. Pneumonia was excluded in the absence of an infiltrate on chest imaging, other (respiratory) diagnosis, or pneumonia which developed in participants admitted for another diagnosis (hospital-acquired pneumonia or ventilator-associated pneumonia).

Information on mortality was retrieved from medical records of GPs, hospitals, or nursing homes. Two independent physicians classified the causes of mortality based on the ICD-10 and ICPC coding systems. Coded mortality events were subsequently reviewed by a medical expert in the field. Pneumonia-related mortality was defined as ICD-10 codes J09-J18 and/or ICPC code R81.

Pre-bronchodilator spirometry was performed by trained paramedical personnel at the research center in Ommoord according to the American thoracic society and European respiratory society guidelines (18). FEV_1_, forced vital capacity (FVC), and the FEV_1_/FVC ratio (the Tiffeneau index) were measured with a portable spirometer (SpiroPro, Erich Jaeger GmbH, Hoechberg, Germany). Preserved Ratio Impaired Spirometry (PRISm) was defined as a potentially restrictive lung function with preserved FEV_1_/FVC ratio (FEV_1_ <80% and FEV_1_/FVC ratio ≥70%). Spirometry measurements were available in a random subset of participants at follow-up visits (RS cohorts I-4 and II-2) and at baseline (RS cohort III-1).

### Assessment of Other Covariates

Potential confounders were based on previous literature (19, 20) and their assessment took place at study baseline. Height, weight, and blood pressure were measured at the research center in Ommoord. Body mass index (BMI) was calculated by dividing the body weight (kg) by height squared (m^2^). Blood pressure was measured with a sphygmomanometer at the right arm with the participant in sitting position. The average blood pressure of two consecutive measurements was taken. Hypertension was defined as a blood pressure exceeding 140/90 mmHg or as the use of blood pressure lowering medication. Information on smoking status, pack years, and alcohol consumption was retrieved from questionnaires during home interviews. Smoking status was defined as former, current, or never smoker. Pack years of smoking were calculated by dividing the product of years and daily cigarettes smoked by twenty. Alcohol consumption was reported in g/day and was categorized into none, mild (0-10 g/day), moderate (10-20 g/day), or heavy (>20 g/day). Chronic obstructive pulmonary disease (COPD) cases were based on pre-bronchodilator spirometry or, in the absence of spirometry at the research center, on diagnoses made by respiratory physicians or GPs. Asthma cases were based on physician’s diagnoses in medical records. Type 2 diabetes (DM) was defined as a fasting blood glucose level exceeding 7 mmol/L, a non-fasting blood glucose level exceeding 11.1 mmol/L (in the absence of fasting blood samples), or as the use of antidiabetics. Physical activity was measured according to validated questionnaires and expressed in metabolic equivalent of task (MET) hours/week. Serum C-reactive protein (CRP) levels were measured with an immunoturbidimetric assay and reported in mg/L. Use of medication that may influence serum Ig levels and/or pneumonia risk was retrieved from linkage with computerized records of pharmacies in the study district and comprised pneumococcal vaccines, inhaled or oral corticosteroids, proton pump inhibitors, angiotensin converting enzyme inhibitors, or antiepileptic or antipsychotic drugs (21–23).

### Statistical Analyses

We performed binomial logistic regression analyses to obtain odds ratios (ORs) and 95% confidence intervals (95% CIs) for the association between serum Igs and prevalent pneumonia. Three models were applied. A first model was adjusted for age and sex. A second model was adjusted for smoking status, pack years, and alcohol consumption additional to the first model. A third model included confounders that could also act as mediators and comprised BMI, DM, asthma, COPD, hypertension, physical activity, and CRP additional to the first two models.

We performed Cox proportional hazards regression analyses to obtain hazard ratios (HRs) and 95% CIs for the association of serum Igs with risk of pneumonia-related mortality. The proportional hazards assumption was checked through the Schoenfeld test. Participants were followed until pneumonia-related mortality, other mortality, loss to follow-up, or January 1^st^ 2015, whichever came first. Three models were applied for the Cox analyses. In a first model we adjusted for age, sex, and RS cohort. A second model additionally included smoking status, pack years, and alcohol consumption. A third model included BMI, DM, asthma, COPD, hypertension, and CRP additional to the first two models.

After exclusion of participants with prevalent pneumonia, we performed Cox proportional hazards regression analyses to obtain HRs and 95% CIs for the association between serum Igs and incident pneumonia. The proportional hazards assumption was checked through the Schoenfeld test. Participants were followed until first incident pneumonia event, death, loss to follow-up, or end of follow-up (May 17^th^ 2018), whichever occurred first. Three models were constructed including the same covariates as the models for prevalent pneumonia with the addition of RS cohort to take temporal trends into account. We performed a sensitivity analysis by adding PRISm as potential confounder based on biological plausibility to the third model. Furthermore, a sensitivity analysis was performed after exclusion of participants with Ig levels outside the reference range and users of potentially immunomodulating medication to exclude the influence of transient fluctuations in serum Ig levels. For comparison, we applied both the manufacturer’s recommended and our own calculated reference ranges in this sensitivity analysis. We furthermore performed a sensitivity analysis after exclusion of probable incident pneumonia events and pneumonia hospitalizations that could not be verified. Predefined stratifications were performed by age (cut-off 65 years) and sex.

We performed negative binomial regression analyses to obtain incidence rate ratios (IRRs) and 95% CIs for the association between serum Igs and the number of incident pneumonia events. There was no zero inflation in any model. Three models were applied adjusting for the same covariates as in the binomial logistic regression analyses with prevalent pneumonia.

We performed linear regression analyses to obtain betas and 95% CIs for the association of serum Igs with FEV_1_, FVC, and the FEV_1_/FVC ratio. There was no heteroscedasticity. Linearity was checked with ordinary least squares regression analyses with three knots. We applied three models. In a first model, we adjusted for age, sex, and time interval between blood drawing and spirometry. In a second model, we additionally adjusted for smoking status and pack years. A third model included BMI, DM, hypertension, and physical activity additional to the first two models. Analyses were furthermore stratified by asthma and COPD status. For the association of serum Igs with PRISm and COPD, we performed binomial logistic regression analyses applying the same three models as for the linear regression analyses with lung function.

Results of all analyses were presented per unit (g/L) increase in serum Ig levels. Missing values in covariates were imputed with multivariate imputation by chained equations (6 imputations, 10 iterations). Convergence was reached and the distribution of covariates before and after imputation was similar. Missingness was ≤2% for all covariates, except for physical activity and alcohol consumption (14.1% and 20.4% respectively). All analyses were performed with R Statistical Software version 4.0.2.

## Results

### Study Population Characteristics

We included 8,766 participants with a median age of 62.2 years and of whom 57% were women. Almost half our study population (n = 4,152) comprised former smokers and 515 participants (5.9%) had COPD. A total of 398 participants (4.5%) had asthma and 231 (2.6%) had PRISm. Median levels of serum Igs were 2.10 g/L for IgA, 9.70 g/L for IgG, and 0.85 g/L for IgM. Baseline characteristics of the study population are shown in **Table 1**.

**Table 1:** Baseline characteristics of 8,766 participants with immunoglobulin measurements and informed consent for follow-up.

Sex, female, n (%)	4,994 (57.0)
Age, years, median (IQR)	62.2 (57.4-70.7)
Serum IgA, g/L, median (IQR)	2.10 (1.57-2.78)
Serum IgG, g/L, median (IQR)	9.70 (8.30-11.20)
Serum IgM, g/L, median (IQR)	0.85 (0.59-1.23)
BMI, kg/m^2^, median (IQR)	26.8 (24.5-29.6)
Hypertension, n (%)	5,345 (61.0)
Diabetes mellitus, n (%)	1,041 (11.9)
Asthma, n (%)	398 (4.5)
COPD, n (%)	515 (5.9)
Smoking status, n (%)	
- Never - Former - Current	- 2,907 (33.2) - 4,152 (47.4) - 1,707 (19.5)
Pack years, median (IQR)	6.0 (0.0-25.0)
Alcohol consumption, n (%)	
- None - Mild (0-10 g/day) - Moderate (10-20 g/day) - Heavy (>20 g/day)	- 1,532 (17.5) - 4,716 (53.8) - 1,447 (16.5) - 1,071 (12.2)
Standardized physical activity, MET hours/week, median (IQR)	-0.16 (-0.67-0.53)
Use of immunomodulating medication^*^, n (%)	1,576 (18.0)
Serum CRP, mg/L, median (IQR)	1.50 (0.60-3.40)

IQR, interquartile range; BMI, body mass index; COPD, chronic obstructive pulmonary disease; MET, metabolic equivalent of task; CRP, C-reactive protein.

^*^Comprises pneumococcal vaccines, inhaled or oral corticosteroids, proton pump inhibitors, angiotensin converting enzyme inhibitors, antiepileptic or antipsychotic drugs.

A total of 43 participants (0.5%) had prevalent pneumonia. Of those participants without a history of pneumonia at baseline, 428 (4.9%) developed pneumonia during a median follow-up of 9.8 years (interquartile range [IQR]: 7.4-15.3). In participants that developed recurrent pneumonias (n=75), the median number of events was 2 (IQR: 2-3). During a median follow-up of 8.6 years (IQR: 7.0-14.2), 78 (0.9%) participants had died of pneumonia.

### Association of Serum Igs With Prevalent Pneumonia, Incident Pneumonia, and Pneumonia-Related Mortality

Higher serum IgA levels were associated with an increased odds of prevalent pneumonia, but this did not reach statistical significance (OR: 1.18; 95% CI: 0.94-1.47). We did not find associations of serum IgG and IgM with prevalent pneumonia (**Table 2**).

**Table 2:** Association between serum immunoglobulins and prevalent pneumonia.

	N events/total	Odds Ratio (95% Confidence Interval)		
		*Model 1*	*Model 2*	*Model 3*
**IgA**	43/8,765	1.16 (0.93-1.43)	1.18 (0.95-1.46)	1.18 (0.94-1.47)
**IgG**	43/8,755	1.01 (0.89-1.14)	1.02 (0.90-1.15)	1.03 (0.91-1.16)
**IgM**	43/8,761	1.03 (0.84-1.27)	1.04 (0.85-1.27)	1.03 (0.83-1.28)

Model 1 was adjusted for age and sex; Model 2 was adjusted for model 1, smoking status, pack years, and alcohol consumption; Model 3 was adjusted for model 2, BMI, DM, asthma, COPD, hypertension, physical activity, and serum CRP.

N events/total refers to the number of participants with prevalent pneumonia and the total number of participants included in the analyses.

IgA/IgG/IgM, immunoglobulin A/G/M; BMI, body mass index; DM, diabetes mellitus; COPD, chronic obstructive pulmonary disease; CRP, C-reactive protein.

Serum IgA and IgM were not associated with pneumonia-related mortality. However, higher serum IgG levels were associated with an increased risk of pneumonia-related mortality (HR: 1.08; 95% CI: 1.01-1.16) (**Table 3**).

**Table 3:** Association between serum immunoglobulins and risk of pneumonia-related mortality.

	N events/total	Hazard Ratio (95% Confidence Interval)		
		*Model 1*	*Model 2*	*Model 3*
**IgA**	78/8,765	0.88 (0.71-1.10)	0.89 (0.72-1.11)	0.88 (0.71-1.09)
**IgG**	78/8,755	**1.08 (1.00-1.16)**	**1.08 (1.01-1.16)**	**1.08 (1.01-1.16)**
**IgM**	78/8,761	1.01 (0.87-1.16)	1.00 (0.87-1.16)	1.00 (0.87-1.16)

Model 1 was adjusted for age, sex, and Rotterdam Study cohort; Model 2 was adjusted for model 1, smoking status, pack years, and alcohol consumption. Model 3 was adjusted for model 2, BMI, DM, asthma, COPD, hypertension, and serum CRP.

N events/total refers to the number of participants that died from pneumonia and the total number of participants included in the analyses.

Significant associations (P <0.05) are in bold.

IgA/IgG/IgM, immunoglobulin A/G/M; BMI, body mass index; DM, diabetes mellitus; COPD, chronic obstructive pulmonary disease; CRP, C-reactive protein.

**Figure 1:**
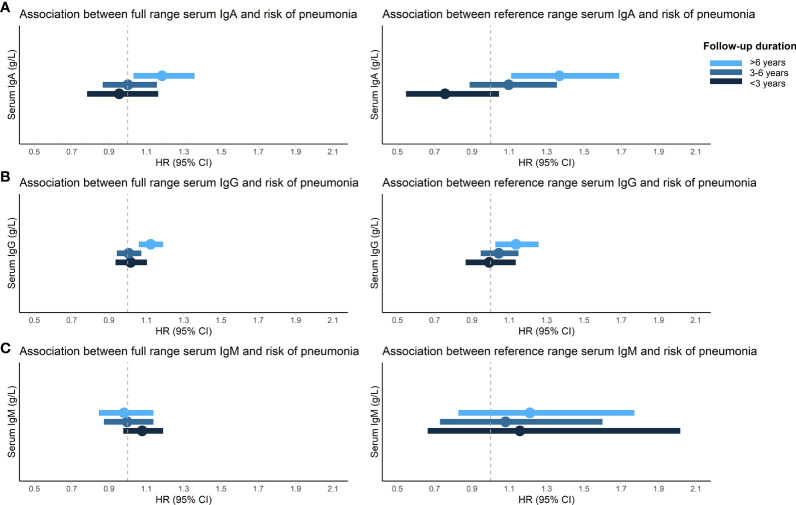
Association between serum immunoglobulins and risk of incident pneumonia. Displayed are the associations of IgA **(A)**, IgG **(B)**, and IgM **(C)** with risk of incident pneumonia stratified by follow-up duration. HRs (dots) and 95% CIs (horizontal bars) are displayed for both the full and reference range of serum immunoglobulins. Reference range was defined as 2.5^th^-97.5^th^ percentiles of this population (0.86-4.76 g/L for IgA, 6.20-15.10 g/L for IgG, and 0.28-2.64 g/L for IgM) and exclusion of medication use that can influence serum immunoglobulin levels and/or pneumonia risk (pneumococcal vaccines, inhaled or oral corticosteroids, proton pump inhibitors, angiotensin converting enzyme inhibitors, antiepileptic or antipsychotic drugs). HRs are adjusted for age, sex, Rotterdam Study cohort, smoking status, pack years, and alcohol consumption. IgA/IgG/IgM, immunoglobulin A/G/M; HR, hazard ratio; 95% CI, 95% confidence interval.

The proportional hazards assumption was violated for the association between Igs and incident pneumonia and therefore the follow-up time was divided into three strata (<3 years, 3-6 years, >6 years) in which the assumption held. After six years of follow-up, higher IgA (HR: 1.15; 95% CI: 1.00-1.32) and IgG (HR: 1.13; 95% CI: 1.06-1.19) levels were associated with an increased risk of incident pneumonia. Serum IgM was not associated with incident pneumonia. Moreover, there were no associations between serum Igs and incident pneumonia in the first six years of follow-up (**Table 4**). After exclusion of participants with Ig levels outside the reference range and users of potentially immunomodulating medication, results were comparable. Most notably, the association between serum IgA and incident pneumonia risk after six years of follow-up became stronger (HR for reference range based on percentiles: 1.36; 95% CI: 1.10-1.68) (**Supplementary Table S1** and **Figure 1**). Generally, results for reference range analyses based on our calculated reference ranges and based on assay manufacturer’s recommendations were comparable (**Supplementary Table S1**). Additional adjustment for PRISm yielded similar results for the association between serum Igs and incident pneumonia (**Supplementary Table S2**). When only definite incident pneumonia cases were included as outcome effect estimates remained similar, although the association with IgA after six years of follow-up had lost significance (HR: 1.11; 95% CI: 0.95-1.30) (**Supplementary Table S3**). However, within the reference range and after exclusion of users of potentially immunomodulating medication, both IgA (HR: 1.34; 95% CI: 1.06-1.70) and IgG (HR: 1.13; 95% CI: 1.01-1.26) were associated with an increased risk of definite incident pneumonia after six years of follow-up (**Supplementary Table S4**). Within the first six years of follow-up, no differential effects of age or sex were noted (data not shown). After six years of follow-up, effect estimates were also comparable for men and women. For age, higher serum IgA levels were associated with an increased risk of incident pneumonia in participants ≤65 years (HR: 1.26; 95% CI: 1.06-1.50), whereas this was not observed in participants >65 years. However, interactions by age or sex were not statistically significant (**Supplementary Table S5**). Higher serum IgG levels were associated with an increased incident pneumonia risk regardless of age or sex (**Supplementary Table S5**). In addition, every g/L increase in serum IgG was associated with a 4% higher number of incident pneumonia events (IRR: 1.04; 95% CI: 1.00-1.09) (**Supplementary Table S6**).

**Table 4:** Association between serum immunoglobulins and risk of incident pneumonia.

		N events/total	Hazard Ratio (95% Confidence Interval)		
Follow-up time			*Model 1*	*Model 2*	*Model 3*
*0-3 years*	**IgA**	92/8,722	0.95 (0.78-1.16)	0.95 (0.78-1.16)	0.94 (0.77-1.15)
	**IgG**	92/8,712	1.01 (0.93-1.10)	1.02 (0.93-1.10)	1.02 (0.94-1.10)
	**IgM**	92/8,718	1.08 (0.98-1.19)	1.08 (0.98-1.19)	1.08 (0.98-1.19)
*3-6 years*	**IgA**	176/8,255	0.97 (0.84-1.12)	1.00 (0.87-1.16)	0.98 (0.84-1.13)
	**IgG**	176/8,245	0.99 (0.92-1.05)	1.01 (0.94-1.07)	1.00 (0.94-1.07)
	**IgM**	175/8,251	1.00 (0.87-1.14)	1.00 (0.87-1.14)	1.00 (0.87-1.14)
*>6 years*	**IgA**	160/7,546	**1.15 (1.00-1.32)**	**1.18 (1.03-1.36)**	**1.15 (1.00-1.32)**
	**IgG**	160/7,536	**1.10 (1.04-1.17)**	**1.12 (1.06-1.19)**	**1.13 (1.06-1.19)**
	**IgM**	160/7,543	0.98 (0.84-1.15)	0.98 (0.85-1.14)	0.98 (0.85-1.14)

Model 1 was adjusted for age, sex, and Rotterdam Study cohort; Model 2 was adjusted for model 1, smoking status, pack years, and alcohol consumption; Model 3 was adjusted for model 2, BMI, DM, asthma, COPD, hypertension, physical activity, and serum CRP.

N events/total refers to the number of participants with incident pneumonia and the total number of participants included in the analyses.

Significant associations (P <0.05) are in bold.

IgA/IgG/IgM, immunoglobulin A/G/M; BMI, body mass index; DM, diabetes mellitus; COPD, chronic obstructive pulmonary disease; CRP, C-reactive protein.

### Association Between Serum Igs and Lung Function Values or Spirometry Patterns

**Figure 2:**
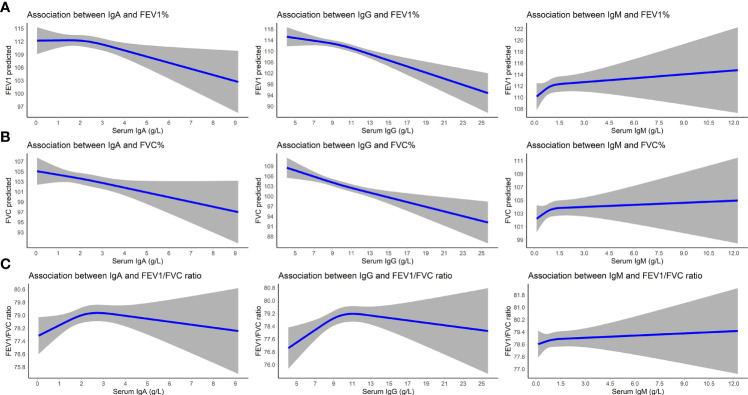
Association between serum immunoglobulins and lung function. Displayed are betas (lines) and 95% CIs (gray areas) for the associations of immunoglobulins with FEV_1_ predicted **(A)**, FVC predicted **(B)**, and the FEV_1_/FVC ratio **(C)**. Betas are adjusted for age, sex, time interval between blood drawing and spirometry, smoking status, and pack years. IgA/IgG/IgM, immunoglobulin A/G/M; 95% CI, 95% confidence interval; FEV_1_, forced expiratory volume in one second; FVC, forced vital capacity.

Higher serum IgA and IgG levels were associated with lower FEV_1_ and FVC values (betas between -0.85 and -0.69% predicted). Serum IgM was not associated with lung function values. IgG displayed an inverse U-shaped association with the FEV_1_/FVC ratio, although not significantly (**Table 5** and **Figure 2**). We furthermore found an increased odds of PRISm with higher IgA (OR: 1.14; 95% CI: 1.00-1.31) and IgG levels (OR: 1.11; 95% CI: 1.05-1.17), while none of the Igs was associated with COPD (**Table 5**).

**Table 5:** Association between serum immunoglobulins and lung function values or spirometry patterns.

FEV_1_% predicted				
	**Beta (95% Confidence Interval)**			
	*Total N*	*Model 1*	*Model 2*	*Model 3*
**IgA**	3,960	-0.35 (-1.03 – 0.34)	**-0.88 (-1.54 – -0.22)**	-0.76 (-1.42 – -0.10)
**IgG**	3,957	-0.45 (-0.74 – -0.15)^*^	**-0.88 (-1.16 – -0.59)**	-0.85 (-1.14 – -0.57)
**IgM**	3,960	0.50 (-0.13 – 1.14)	0.43 (-0.18 – 1.04)	0.37 (-0.24 – 0.97)
**FVC % predicted**				
		**Beta (95% Confidence Interval)**		
	*Total N*	*Model 1*	*Model 2*	*Model 3*
**IgA**	3,960	**-0.59 (-1.17 – -0.01)**	**-0.87 (-1.44 – -0.30)**	-0.69 (-1.25 – -0.12)
**IgG**	3,957	**-0.55 (-0.80 – -0.30)**	**-0.78 (-1.03 – -0.53)**	-0.73 (-0.98 – -0.49)
**IgM**	3,960	0.30 (-0.23 – 0.84)	0.27 (-0.26 – 0.79)	0.18 (-0.34 – 0.70)
**FEV_1_/FVC ratio (Tiffeneau index)**				
		**Beta (95% Confidence Interval)**		
	*Total N*	*Model 1*	*Model 2*	*Model 3*
**IgA**	3,960	0.32 (0.06 – 0.57)^*^	0.13 (-0.11 – 0.38)	0.08 (-0.16 – 0.32)
**IgG**	3,957	0.24 (0.14 – 0.35)^*^	0.10 (-0.00 – 0.21)^*^	0.09 (-0.02 – 0.19)^*^
**IgM**	3,960	0.10 (-0.13 – 0.34)	0.08 (-0.15 – 0.30)	0.10 (-0.13 – 0.32)
**PRISm**				
			**Odds Ratio (95% Confidence Interval)**	
	*Total N*	*Model 1*	*Model 2*	*Model 3*
**IgA**	3,960	1.13 (0.99 – 1.28)	**1.17 (1.03 – 1.33)**	1.14 (1.00 – 1.31)
**IgG**	3,957	**1.09 (1.03 – 1.15)**	**1.11 (1.05 – 1.18)**	1.11 (1.05 – 1.17)
**IgM**	3,960	0.90 (0.74 – 1.09)	0.90 (0.74 – 1.10)	0.92 (0.75 – 1.11)
**COPD**				
			**Odds Ratio (95% Confidence Interval)**	
	*Total N*	*Model 1*	*Model 2*	*Model 3*
**IgA**	8,765	1.00 (0.87-1.16)	1.07 (0.92-1.24)	1.07 (0.93-1.24)
**IgG**	8,755	**0.90 (0.84-0.97)**	0.94 (0.88-1.01)	0.94 (0.88-1.01)
**IgM**	8,761	0.99 (0.88-1.12)	1.00 (0.88-1.13)	1.00 (0.88-1.13)

^*^Non-linear association.

Model 1 was adjusted for age, sex, and time interval between blood drawing and spirometry; Model 2 was adjusted for model 1, smoking status, and pack years; Model 3 was adjusted for model 2, BMI, DM, hypertension, and physical activity.

Significant associations (P <0.05) are in bold.

IgA/IgG/IgM, immunoglobulin A/G/M; FEV_1_, forced expiratory volume in one second; FVC, forced vital capacity; PRISm, preserved ratio impaired spirometry; COPD, chronic obstructive pulmonary disease; BMI, body mass index; DM, diabetes mellitus.

In the stratified analyses, higher serum IgG levels were associated with lower FEV_1_ and FVC values in participants with COPD (beta for FEV_1_: -1.35; 95% CI: -2.49 – -0.21% predicted; beta for FVC: -1.31; 95% CI: -2.46 – -0.16% predicted). Serum Igs were not associated with the FEV_1_/FVC ratio, regardless of asthma or COPD status (**Supplementary Table S7**).

## Discussion

In our population-based cohort of middle-aged and older individuals, higher serum IgA and IgG levels were associated with an increased risk of incident pneumonia after long-term follow-up and with lower FEV_1_ and FVC predicted values, as well as an increased odds of PRISm. Furthermore, higher serum IgG levels were associated with an increased risk of recurrent incident pneumonia and pneumonia-related mortality.

Most previous studies described an association between low serum Ig levels and (severe) pneumonia, although these studies were performed in other populations, including younger and/or immunodeficient individuals. One study in immunocompetent individuals reported no baseline difference in mean IgA, IgG, or IgM levels between pneumonia patients and age- and sex-matched controls (12). Another cross-sectional study reported higher Ig levels, with only IgA reaching statistical significance, in CAP patients that died within a month after discharge compared to the patients that survived (16). To the best of our knowledge, longitudinal data on the association between serum Igs and pneumonia risk in a general population was lacking. Possible explanations for our findings include chronic inflammation, gammopathy or decreased functionality of serum Igs, pulmonary fibrosis, or reverse causation.

Previously we have shown that, after an approximate age of 60-65 years, higher age was linearly associated with higher serum IgA and IgG levels in our study cohort (11). This implicates that higher serum Igs at older age may reflect a chronic low-grade immune activation, i.e. ‘inflammaging’, associated with increased morbidity and mortality (24). Mostly elevated levels of pro-inflammatory cytokines have been described in inflammaging, but recently Igs have been considered a marker of inflammaging as well. IgG loses its galactosylation at older age increasing its pro-inflammatory potential (24, 25). In a study of four different European populations, age-related changes in IgG glycosylation strongly correlated with various physiological traits, including a lower FEV_1_, FVC, and peak expiratory flow (26). As containment of the immune response is essential in order to prevent severe outcomes in pneumonia, previous studies have linked chronic inflammation to pneumonia susceptibility (1, 9). In a prospective, population-based cohort of American elderly, higher baseline levels of tumor necrosis factor alpha (TNF-α) and interleukin 6 (IL-6) were associated with an increased pneumonia risk during follow-up, even after adjustment for traditional risk factors and comorbidities (27). In mouse models, increased TNF-α levels have been shown to impair monocyte function (28). *In vitro* treatment of cells with pro-inflammatory cytokines increased adhesion of *Streptococcus Pneumoniae* (*S. Pneumoniae*) to host cells hundred-fold (8). TNF-α infusion in mice furthermore increased pulmonary levels of polymeric Ig receptor (pIgR), a receptor commonly involved in mucosal transport of IgA, but also capable of increasing susceptibility to *S. Pneumoniae* (8, 29). Although serum Igs have not been studied as extensively as cytokines in the pathophysiology of pneumonia, elevated levels of serum Igs may be associated with an increased risk of developing pneumonia through similar pathways. We only reported an increased pneumonia risk after long-term follow-up, which may be due to requirement of prolonged inflammation in order to reach a threshold for clinically overt disease. Future research is warranted to explore this hypothesis.

Higher serum IgA and IgG levels at older age may also signify monoclonal or polyclonal gammopathy (30, 31). This could imply that these elevated Ig levels may be less functional (i.e. may protect less against infections). In a Swedish population-based study, monoclonal gammopathy of undetermined significance was associated with an increased risk of viral and bacterial infections (including pneumonia) over a follow-up period of 5-10 years (32). This hypothesis seems less probable in our cohort, since reference range analyses yielded similar or even larger effect estimates for the association with pneumonia risk. However, even in the absence of an underlying gammopathy, serum Igs at older age could be less functional and less adequate in prevention against infections. Previous case-control studies reported that IgG antibody levels after pneumococcal vaccines were comparable for most *S. Pneumoniae* serotypes in older compared to younger individuals, while the functional antibody activity was lower in older individuals (33, 34). Within the RS, we did not have information on functionality of Igs or vaccine responses to explore the avidity of the measured Igs.

Interestingly, higher IgA levels were stronger associated with pneumonia risk than IgG, particularly within the reference range. This may be explained by the fact that IgA is the most abundant Ig in the respiratory tract (35). Therefore, inflammation involving IgA may be stronger associated with pulmonary outcomes. Although we included serum measurements in our analyses, IgA in lung secretions is partly obtained from the blood stream by transudation so higher serum levels may indicate higher pulmonary levels as well (35). Conversely, we reported associations of serum IgG rather than IgA with increased incident pneumonia count and risk of pneumonia-related mortality. IgG, being the most prevalent Ig in the serum (35), may denote more severe or systemic inflammation subsequently being associated with repeated and possibly fatal events. This is supported by the findings of an American population-based study reporting highest mortality risks (including infectious mortality) with higher IgG rather than IgA or IgM levels (36).

Higher serum IgA and IgG levels were associated with lower FEV_1_ and FVC predicted, and an increased odds of PRISm. No associations were reported with the FEV_1_/FVC ratio or COPD, suggesting that Igs may be associated with a restrictive and not an obstructive spirometry pattern (37). This is in contrast to previous studies that report an association of low levels of serum IgA and IgG with COPD exacerbations. However, these studies were exclusively performed in COPD patients instead of in the general population, had smaller sample sizes, and had categorized serum Ig levels which may have resulted in loss of information (38, 39). One of the well-known causes of lung restriction is pulmonary fibrosis (37). Aging is associated with changes in pulmonary fibroblasts and extracellular matrix (ECM) that stimulate pulmonary fibrosis and reduce lung elasticity (40, 41). Transforming growth factor beta (TGF-β) is a key player in pulmonary fibrosis by promoting differentiation of myofibroblasts (leading to ECM production) and inhibiting anti-fibrotic molecules and growth and repair of alveolar epithelial cells (42). However, TGF-β is also involved in Ig class switching resulting in increased levels of IgA and IgG2b in mice (43). For IgG, the association with lower FEV_1_ and FVC predicted was strongest in participants with COPD. This may be explained by autoantibodies, as previously anti-tissue antibodies have been associated with a worse FEV_1_ and carbon monoxide diffusion capacity in COPD patients (44). Pre-existing pulmonary fibrosis may also induce elevated Ig levels and an increased risk of developing pneumonia. Idiopathic pulmonary fibrosis patients display a less diverse pulmonary microbiome with higher levels of certain bacteria compared to healthy controls. This is associated with elevated levels of pro-inflammatory cytokines, alveolar inflammation, and unfavorable clinical outcomes (45). We did not have pulmonary fibrosis cases in our cohort to compare the association of serum Igs with incident pneumonia in participants with and without pulmonary fibrosis. Future studies should investigate whether serum Ig levels may mediate an association between pulmonary fibrosis and pneumonia. However, it should be noted that PRISm does not always indicate (the development of) lung restriction/pulmonary fibrosis (46) and that future studies are warranted to explore the association between serum Igs and lung function changes over time.

Although we adjusted for a wide range of potential confounders, it should be borne in mind that elevated serum Igs may reflect underlying unmeasured or unknown conditions associated with an increased pneumonia risk. Lastly, it is possible that our findings are false positive. However, this does not seem likely since we reported consistent results for all investigated pulmonary outcomes and throughout sensitivity and stratified analyses.

Important strengths of our study include the population-based design, inclusion of a wide range of potential confounders and medication use, and the long-term follow-up with elaborate ascertainment of pneumonia events. We furthermore had access to standardized spirometry measurements. However, we did not have longitudinal measurements of serum Igs and were therefore unable to investigate the association of serum Ig levels over time with incident pneumonia and lung function. We also did not have information on Ig glycosylation profiles or functionality, gammopathy, or pulmonary fibrosis which may have aided in understanding the involved pathophysiology. Future research is warranted to replicate our findings, although we are not aware of a similarly large prospective cohort with the required data. If results are validated, the pathophysiological mechanisms underlying the reported associations need to be elucidated.

## Data Availability Statement

The datasets presented in this article are not readily available because data cannot be made freely available in a public repository due to restrictions based on privacy regulations and informed consent of the participants. Requests to access the datasets should be directed to the management team of the Rotterdam Study (datamanagement.ergo@erasmusmc.nl).

## Ethics Statement

The studies involving human participants were reviewed and approved by the Medical Ethics Committee of the Erasmus MC (registration number MEC 02.1015) and the Dutch Ministry of Health, Welfare and Sport (Population Screening Act WBO, license number 1071272-159521-PG). The patients/participants provided their written informed consent to participate in this study.

## Author Contributions

SK, AV, LL, RP, PvH, GB, LC, and VD contributed to study design and data interpretation. AV and LL contributed to data collection. SK contributed to data curation, data analysis and writing of the manuscript. AV, LL, RP, PvH, GB, LC, and VD critically reviewed the manuscript. All authors approved the final version of the manuscript and agreed to be accountable for all aspects of the work.

## Funding

This work was supported by Takeda Pharmaceutical Company Limited [grant number IIR-NLD-002671 to V.A.S.H. Dalm]. The funder was not involved in the study design, collection, analysis, interpretation of data, the writing of this article or the decision to submit it for publication. The Rotterdam Study was supported by the Erasmus Medical Center, Erasmus University Rotterdam, the Netherlands Organization for Scientific Research (NWO), the Netherlands Organisation for Health Research and Development (ZonMw), the Netherlands Heart Foundation, the Research Institute for Diseases in the Elderly (RIDE); the Netherlands Genomics Initiative (NGI), the Ministry of Education, Culture and Science; the Ministry of Health Welfare and Sports; the European Commission (DG XII); and the Municipality of Rotterdam. Lung research was financially supported by the Fund for Scientific Research Flanders [grant number 3G037618].

## Conflict of Interest

The authors declare that the research was conducted in the absence of any commercial or financial relationships that could be construed as a potential conflict of interest.

## Publisher’s Note

All claims expressed in this article are solely those of the authors and do not necessarily represent those of their affiliated organizations, or those of the publisher, the editors and the reviewers. Any product that may be evaluated in this article, or claim that may be made by its manufacturer, is not guaranteed or endorsed by the publisher.
