# Supplementary material for: Serum Immunoglobulins, Pneumonia Risk, and Lung Function in Middle-Aged and Older Individuals: A Population-Based Cohort Study
Source: Front Immunol. 2022 Jun 2;13:868973. doi: 10.3389/fimmu.2022.868973 (PMC9215210; doi:10.3389/fimmu.2022.868973)
Supplement: Supplementary file 1 [file Table_1.docx]

| **Supplementary Table S1. Association between reference range serum immunoglobulins and risk of incident pneumonia** | | | | | |
| --- | --- | --- | --- | --- | --- |
| **Calculated reference range^*^** | | | | | |
|  |  |  | **Hazard Ratio (95% Confidence Interval)** | | |
| **Follow-up time** |  | *N events/total* | *Model 1* | *Model 2* | *Model 3* |
| *0-3 years* | **IgA** | 63/6,825 | 0.75 (0.54-1.03) | 0.76 (0.55-1.04) | 0.75 (0.54-1.03) |
|  | **IgG** | 63/6,820 | 0.98 (0.86-1.12) | 0.99 (0.87-1.13) | 0.99 (0.87-1.14) |
|  | **IgM** | 60/6,849 | 1.14 (0.65-1.98) | 1.16 (0.66-2.02) | 1.15 (0.66-2.01) |
| *3-6 years* | **IgA** | 120/6,506 | 1.05 (0.85-1.29) | 1.10 (0.89-1.36) | 1.10 (0.88-1.36) |
|  | **IgG** | 122/6,511 | 1.02 (0.93-1.12) | 1.04 (0.95-1.15) | 1.04 (0.94-1.14) |
|  | **IgM** | 119/6,529 | 1.06 (0.71-1.57) | 1.08 (0.73-1.60) | 1.07 (0.72-1.58) |
| *>6 years* | **IgA** | 109/6,016 | **1.30 (1.05-1.60)** | **1.37 (1.11-1.69)** | **1.36 (1.10-1.68)** |
|  | **IgG** | 108/6,017 | 1.10 (1.00-1.22) | **1.14 (1.03-1.26)** | **1.14 (1.03-1.26)** |
|  | **IgM** | 113/6,040 | 1.22 (0.83-1.80) | 1.21 (0.83-1.77) | 1.23 (0.84-1.81) |
| **Assay recommended reference range^*^** | | | | | |
|  |  |  | **Hazard Ratio (95% Confidence Interval)** | | |
| **Follow-up time** |  | *N events/total* | *Model 1* | *Model 2* | *Model 3* |
| *0-3 years* | **IgA** | 62/6,648 | 0.81 (0.57-1.15) | 0.82 (0.58-1.16) | 0.81 (0.57-1.15) |
|  | **IgG** | 63/6,595 | 0.96 (0.84-1.10) | 0.97 (0.85-1.11) | 0.97 (0.85-1.11) |
|  | **IgM** | 54/6,330 | 1.00 (0.51-1.96) | 1.02 (0.52-2.00) | 1.01 (0.52-1.99) |
| *3-6 years* | **IgA** | 116/6,350 | 1.08 (0.85-1.38) | 1.13 (0.89-1.45) | 1.13 (0.88-1.44) |
|  | **IgG** | 119/6,293 | 1.01 (0.92-1.11) | 1.03 (0.93-1.14) | 1.02 (0.93-1.13) |
|  | **IgM** | 114/6,042 | 1.11 (0.71-1.74) | 1.13 (0.73-1.77) | 1.13 (0.73-1.76) |
| *>6 years* | **IgA** | 104/5,879 | **1.49 (1.16-1.91)** | **1.56 (1.21-2.01)** | **1.55 (1.20-2.00)** |
|  | **IgG** | 106/5,807 | 1.07 (0.97-1.18) | 1.10 (0.99-1.22) | 1.10 (0.99-1.22) |
|  | **IgM** | 106/5,597 | 1.04 (0.65-1.66) | 1.05 (0.66-1.67) | 1.07 (0.67-1.70) |
| ^*^Comprises reference range values of serum immunoglobulins and exclusion of medication use that can influence serum immunoglobulin levels and/or pneumonia risk (pneumococcal vaccines, inhaled or oral corticosteroids, proton pump inhibitors, angiotensin converting enzyme inhibitors, antiepileptic or antipsychotic drugs). Calculated reference ranges (based on 2.5^th^-97.5^th^ percentiles of this population) were 0.86-4.76 g/L for IgA, 6.20-15.10 g/L for IgG, and 0.28-2.64 g/L for IgM. Assay recommended reference ranges were 0.7-4.0 g/L for IgA, 7.0-16.0 g/L for IgG, and 0.4-2.3 g/L for IgM.  Model 1 was adjusted for age, sex, and Rotterdam Study cohort; Model 2 was adjusted for model 1, smoking status, pack years, and alcohol consumption; Model 3 was adjusted for model 2, BMI, DM, asthma, COPD, hypertension, physical activity, and serum CRP.  N events/total refers to the number of participants with incident pneumonia and the total number of participants included in the analyses.  Significant associations (P <0.05) are in bold.  IgA/IgG/IgM, immunoglobulin A/G/M; BMI, body mass index; DM, diabetes mellitus; COPD, chronic obstructive pulmonary disease; CRP, C-reactive protein. | | | | | |

| **Supplementary Table S2. Association between serum immunoglobulins and risk of incident pneumonia additionally adjusted for PRISm** | | | | |
| --- | --- | --- | --- | --- |
|  |  |  | **Hazard Ratio (95% Confidence Interval)** | |
| **Follow-up time** |  | *N events/total* | *Fully adjusted model* | *Fully adjusted model + PRISm* |
| *0-3 years* | **IgA** | 92/8,722 | 0.94 (0.77-1.15) | 0.93 (0.76-1.14) |
|  | **IgG** | 92/8,712 | 1.02 (0.94-1.10) | 1.01 (0.93-1.10) |
|  | **IgM** | 92/8,718 | 1.08 (0.98-1.19) | 1.08 (0.98-1.20) |
| *3-6 years* | **IgA** | 176/8,255 | 0.98 (0.84-1.13) | 0.97 (0.84-1.12) |
|  | **IgG** | 176/8,245 | 1.00 (0.94-1.07) | 1.00 (0.94-1.07) |
|  | **IgM** | 175/8,251 | 1.00 (0.87-1.14) | 1.00 (0.87-1.15) |
| *>6 years* | **IgA** | 160/7,546 | **1.15 (1.00-1.32)** | 1.15 (1.00-1.32) |
|  | **IgG** | 160/7,536 | **1.13 (1.06-1.19)** | **1.13 (1.06-1.19)** |
|  | **IgM** | 160/7,543 | 0.98 (0.85-1.14) | 0.99 (0.86-1.14) |
| Fully adjusted model was adjusted for age, sex, Rotterdam Study cohort, smoking status, pack years, alcohol consumption, BMI, DM, asthma, COPD, hypertension, physical activity, and serum CRP.  N events/total refers to the number of participants with incident pneumonia and the total number of participants included in the analyses.  Significant associations (P <0.05) are in bold.  PRISm, preserved ratio impaired spirometry; IgA/IgG/IgM, immunoglobulin A/G/M; BMI, body mass index; DM, diabetes mellitus; COPD, chronic obstructive pulmonary disease; CRP, C-reactive protein. | | | | |

| **Supplementary Table S3. Association between serum immunoglobulins and risk of definite incident pneumonia** | | | | | |
| --- | --- | --- | --- | --- | --- |
|  |  |  | **Hazard Ratio (95% Confidence Interval)** | | |
| **Follow-up time** |  | *N events/total* | *Model 1* | *Model 2* | *Model 3* |
| *0-3 years* | **IgA** | 60/8,722 | 1.03 (0.82-1.29) | 1.04 (0.83-1.30) | 1.01 (0.80-1.27) |
|  | **IgG** | 60/8,712 | 1.03 (0.93-1.14) | 1.03 (0.94-1.14) | 1.03 (0.94-1.14) |
|  | **IgM** | 60/8,718 | 1.08 (0.95-1.21) | 1.08 (0.96-1.22) | 1.08 (0.95-1.21) |
| *3-6 years* | **IgA** | 114/8,255 | 0.90 (0.74-1.09) | 0.93 (0.76-1.12) | 0.90 (0.74-1.09) |
|  | **IgG** | 114/8,245 | 1.00 (0.92-1.08) | 1.02 (0.94-1.11) | 1.02 (0.94-1.10) |
|  | **IgM** | 113/8,251 | 1.05 (0.94-1.18) | 1.05 (0.93-1.17) | 1.05 (0.93-1.18) |
| *>6 years* | **IgA** | 129/7,546 | 1.10 (0.94-1.29) | 1.14 (0.97-1.33) | 1.11 (0.95-1.30) |
|  | **IgG** | 129/7,536 | **1.10 (1.03-1.17)** | **1.12 (1.05-1.19)** | **1.12 (1.05-1.19)** |
|  | **IgM** | 129/7,543 | 0.93 (0.74-1.17) | 0.93 (0.75-1.16) | 0.93 (0.75-1.17) |
| Model 1 was adjusted for age, sex, and Rotterdam Study cohort; Model 2 was adjusted for model 1, smoking status, pack years, and alcohol consumption; Model 3 was adjusted for model 2, BMI, DM, asthma, COPD, hypertension, physical activity, and serum CRP.  N events/total refers to the number of participants with incident pneumonia and the total number of participants included in the analyses.  Significant associations (P <0.05) are in bold.  IgA/IgG/IgM, immunoglobulin A/G/M; BMI, body mass index; DM, diabetes mellitus; COPD, chronic obstructive pulmonary disease; CRP, C-reactive protein. | | | | | |

| **Supplementary Table S4. Association between reference range serum immunoglobulins^*^ and risk of definite incident pneumonia** | | | | | |
| --- | --- | --- | --- | --- | --- |
|  |  |  | **Hazard Ratio (95% Confidence Interval)** | | |
| **Follow-up time** |  | *N events/total* | *Model 1* | *Model 2* | *Model 3* |
| *0-3 years* | **IgA** | 39/6,825 | 0.81 (0.55-1.21) | 0.83 (0.56-1.25) | 0.82 (0.55-1.23) |
|  | **IgG** | 39/6,820 | 0.96 (0.81-1.14) | 0.99 (0.83-1.17) | 0.99 (0.83-1.17) |
|  | **IgM** | 37/6,849 | 1.23 (0.62-2.45) | 1.28 (0.64-2.55) | 1.29 (0.64-2.58) |
| *3-6 years* | **IgA** | 80/6,506 | 1.05 (0.81-1.35) | 1.11 (0.86-1.43) | 1.10 (0.85-1.43) |
|  | **IgG** | 83/6,511 | 1.02 (0.91-1.15) | 1.06 (0.94-1.19) | 1.05 (0.93-1.18) |
|  | **IgM** | 77/6,529 | 1.22 (0.76-1.97) | 1.24 (0.78-1.99) | 1.23 (0.77-1.98) |
| *>6 years* | **IgA** | 88/6,016 | **1.28 (1.01-1.61)** | **1.36 (1.07-1.71)** | **1.34 (1.06-1.70)** |
|  | **IgG** | 89/6,017 | 1.10 (0.98-1.22) | **1.13 (1.01-1.26)** | **1.13 (1.01-1.26)** |
|  | **IgM** | 92/6,040 | 1.11 (0.72-1.72) | 1.11 (0.72-1.70) | 1.11 (0.72-1.72) |
| ^*^Comprises reference range values based on 2.5^th^-97.5^th^ percentiles of this population (0.86-4.76 g/L for IgA, 6.20-15.10 g/L for IgG, and 0.28-2.64 g/L for IgM) and exclusion of medication use that can influence serum immunoglobulin levels and/or pneumonia risk (pneumococcal vaccines, inhaled or oral corticosteroids, proton pump inhibitors, angiotensin converting enzyme inhibitors, antiepileptic or antipsychotic drugs).  Model 1 was adjusted for age, sex, and Rotterdam Study cohort; Model 2 was adjusted for model 1, smoking status, pack years, and alcohol consumption; Model 3 was adjusted for model 2, BMI, DM, asthma, COPD, hypertension, physical activity, and serum CRP.  N events/total refers to the number of participants with incident pneumonia and the total number of participants included in the analyses.  Significant associations (P <0.05) are in bold.  IgA/IgG/IgM, immunoglobulin A/G/M; BMI, body mass index; DM, diabetes mellitus; COPD, chronic obstructive pulmonary disease; CRP, C-reactive protein. | | | | | |

| **Supplementary Table S5. Association between serum immunoglobulins and risk of incident pneumonia stratified by age and sex** | | | | |
| --- | --- | --- | --- | --- |
|  | **Men** | | **Women** | |
|  | *N events/total* | *HR (95% CI)* | *N events/total* | *HR (95% CI)* |
| **IgA** | 91/3,174 | 1.17 (0.98-1.39) | 69/4,372 | 1.07 (0.84-1.36) |
| **IgG** | 91/3,171 | **1.11 (1.01-1.21)** | 69/4,365 | **1.12 (1.04-1.21)** |
| **IgM** | 91/3,173 | 0.99 (0.85-1.15) | 69/4,370 | 0.97 (0.71-1.33) |
|  | **Age ≤65 years** | | **Age >65 years** | |
|  | *N events/total* | *HR (95% CI)* | *N events/total* | *HR (95% CI)* |
| **IgA** | 96/4,849 | **1.26 (1.06-1.50)** | 64/2,697 | 1.04 (0.83-1.30) |
| **IgG** | 96/4,842 | **1.11 (1.03-1.20)** | 64/2,694 | **1.14 (1.04-1.23)** |
| **IgM** | 96/4,847 | 1.00 (0.75-1.32) | 64/2,696 | 0.99 (0.82-1.19) |
| HRs were adjusted for age or sex (depending on the stratification), Rotterdam Study cohort, smoking status, pack years, alcohol consumption, BMI, DM, asthma, COPD, hypertension, physical activity, and serum CRP.  Depicted HRs were for the follow-up stratum of >6 years follow-up.  N events/total refers to the number of participants with incident pneumonia and the total number of participants included in the analyses.  Significant associations (P <0.05) are in bold.  P-values for interaction were >0.10 for all analyses.  IgA/IgG/IgM, immunoglobulin A/G/M; HR, hazard ratio; 95% CI, 95% confidence interval; BMI, body mass index; DM, diabetes mellitus; COPD, chronic obstructive pulmonary disease; CRP, C-reactive protein. | | | | |

| **Supplementary Table S6. Association between serum immunoglobulins and number of incident pneumonia events** | | | |
| --- | --- | --- | --- |
| **All incident pneumonia events** | | | |
|  | **Incidence Rate Ratio (95% Confidence Interval)** | | |
|  | *Model 1* | *Model 2* | *Model 3* |
| **IgA** | 1.03 (0.94-1.13) | 1.05 (0.96-1.15) | 1.03 (0.94-1.13) |
| **IgG** | 1.03 (0.99-1.07) | **1.04 (1.00-1.08)** | **1.04 (1.00-1.09)** |
| **IgM** | 1.01 (0.92-1.10) | 1.00 (0.92-1.10) | 1.01 (0.92-1.10) |
| **Definite incident pneumonia events** | | | |
|  | **Incidence Rate Ratio (95% Confidence Interval)** | | |
|  | *Model 1* | *Model 2* | *Model 3* |
| **IgA** | 1.03 (0.94-1.13) | 1.05 (0.96-1.15) | 1.03 (0.95-1.13) |
| **IgG** | 1.02 (0.99-1.06) | 1.04 (1.00-1.08) | **1.04 (1.00-1.08)** |
| **IgM** | 1.01 (0.92-1.10) | 1.01 (0.92-1.10) | 1.01 (0.92-1.10) |
| Model 1 was adjusted for age and sex; Model 2 was adjusted for model 1, smoking status, pack years, and alcohol consumption; Model 3 was adjusted for model 2, BMI, DM, asthma, COPD, hypertension, physical activity, and serum CRP.  Significant associations (P <0.05) are in bold.  IgA/IgG/IgM, immunoglobulin A/G/M; BMI, body mass index; DM, diabetes mellitus; COPD, chronic obstructive pulmonary disease; CRP, C-reactive protein. | | | |

| **Supplementary Table S7. Association between serum immunoglobulins and lung function values stratified by asthma and COPD status** | | |
| --- | --- | --- |
| **FEV_1_ % predicted** | | |
|  | **Beta (95% Confidence Interval)** | |
|  | *COPD (n=203)* | *Asthma (n=207)* |
| **IgA** | -0.62 (-3.10 – 1.86) | 0.72 (-1.92 – 3.37) |
| **IgG** | **-1.35 (-2.49 – -0.21)** | -1.09 (-2.36 – 0.18) |
| **IgM** | 0.88 (-1.90 – 3.67) | 0.18 (-2.43 – 2.79) |
| **FVC % predicted** | | |
|  | **Beta (95% Confidence Interval)** | |
|  | *COPD (n=203)* | *Asthma (n=207)* |
| **IgA** | -0.72 (-3.22 – 1.78) | 0.83 (-1.53 – 3.19) |
| **IgG** | **-1.31 (-2.46 – -0.16)** | -0.45 (-1.59 – 0.69) |
| **IgM** | 1.51 (-1.28 – 4.30) | 0.23 (-2.10 – 2.57) |
| **FEV_1_/FVC ratio (Tiffeneau index)** | | |
|  | **Beta (95% Confidence Interval)** | |
|  | *COPD (n=203)* | *Asthma (n=207)* |
| **IgA** | 0.09 (-0.81 – 0.99) | 0.09 (-1.00 – 1.18) |
| **IgG** | -0.20 (-0.62 – 0.22) | -0.09 (-0.63 – 0.44) |
| **IgM** | -0.37 (-1.38 – 0.64) | -0.29 (-1.37 – 0.80) |
| Betas are adjusted for age, sex, time interval between blood drawing and spirometry, smoking status, pack years, BMI, DM, hypertension, and physical activity.  Significant associations (P <0.05) are in bold.  P-values for interaction were >0.10 for all analyses.  IgA/IgG/IgM, immunoglobulin A/G/M; FEV_1_, forced expiratory volume in one second; FVC, forced vital capacity; COPD, chronic obstructive pulmonary disease; BMI, body mass index; DM, diabetes mellitus. | | |
